# Anaerobic co-digestion of grass and cow manure: kinetic and GHG calculations

**DOI:** 10.1038/s41598-023-33169-0

**Published:** 2023-04-18

**Authors:** Ayse Hilal Ulukardesler

**Affiliations:** Vocational School of Technical Sciences, Bursa Uludag University, Bursa, Turkey

**Keywords:** Environmental sciences, Chemistry

## Abstract

Grass is a highly desirable substrate for anaerobic digestion because of its higher biodegradability and biogas/methane yield. In this study, anaerobic co-digestion of grass, cow manure and sludge was studied under mesophilic conditions for 65 days. Experiments were performed on a feed ratio of grass/manure from 5 to 25%, respectively. The maximum cumulative biogas and methane yield was obtained as 331.75 mLbiogas/gVS and 206.64 mLCH_4_/gVS for 25% ratio. Also, the results of the experiments were tested on the three different kinetics model which are the first order kinetic model, modified Gompertz model and Logistics model. As a result of the study, it was found that by using grass nearly 480 × 10^6^ kWh/year electricity may be produced and 0.5 × 10^6^ tons/year CO_2_ greenhouse gas emission mitigation may be reached.

## Introduction

Today, energy demand is constantly increasing due to the intense consumption of natural resources such as natural gas, oil and coal in the industries, residences etc.^1^. Being dependent on fossil fuels as primary energy source has caused global climate change, environmental degradation and human health problems^2^. Researchers around the world are concerned about reducing greenhouse gas (GHG) emission corresponding to electricity generation. For this reason, great importance is attached to GHG reduction on the agenda of policy makers. The use of renewable energy sources can positively affect the green energy production and minimize negative effects caused by the use of fossil fuels^3–5^.

Anaerobic digestion (AD) is practiced in various processes and has become an important part of renewable energies. Every year, several million tons of organic waste are disposed by incineration, land applications, landfill, etc. in the world. These wastes are materials such as wood obtained from forests, agriculture wastes and processes of forestry, and wastes of industry, humans, and animals. Microorganisms can consume organic matter through AD by producing biogas consisting of methane (50–70%) and carbon dioxide (30–50%). The use of fossil fuels can be reduced by evaluating the produced methane by cogeneration of heat and electricity or by injection into gas networks^5–8^.

The most common way of using grassland biomass for bioenergy production in Europe is to convert harvested biomass into methane through anaerobic digestion^9^. Interest in the use of grass as a raw material for bioenergy and bio-refining systems is due to its high yield potential in terms of methane production per hectare. The lignin and cellulose content makes it more suitable as energy sources^10^. In spite of the advantages given above, several difficulties must be encountered before using for industrial applications^5^. In order to increase biogas production from lignocellulosic biomass, a pretreatment method is required before further processing. Therefore, the use of lignocellulosic biomass is only economically possible after pretreatment in most cases^11,12^. The biodegradability of lignocellulosic materials can be increased by pretreatment methods. Numerous approaches have been developed and proposed for lignocellulose pretreatments. Pretreatment technologies are generally classified into three categories: physical, chemical and biological, and sometimes combinations of these methods are recommended for more efficient results. Many previous studies have addressed the pretreatment of lignocellulosic biomass^13–15^.

Between physical pretreatment methods milling is proved to be effective and increases hydrolysis efficiency by increasing the specific surface area, by reducing the degree of polymerization and also leading to shearing, This development depends on the type of biomass, the grinding time and grinding type^16,17^. It appears that in biofuel production, the smaller particle size of lignocelluloses yields higher biofuel yields^18^. Nizami^19^ reported that the grinding effect can increase the methane content from 5 to 25%, but higher parasite demands make grinding less attractive. He made sure that pre-processing techniques were suitable for heat treatment such as grass silage, size reduction, and liquid hot water, and that slurry coding could offer more stable processes that were more useful than grass or slurry mono digestion. Tsapekos et al.^5^ investigated the production of biogas from AD of grass using two harvesters, a disc mower and an excavator. According to their study, single digestion of grass with a specific biomethane yield of 329 mLCH_4_/gVS will not guarantee a long-term sustainable energy system. Bedoic et al.^20^ studied residue grass digestion on mono digestion system. They obtained the biochemical methane potential on uncultivated land, river bank highway boundary, between 0.192 and 0.255 Nm^3^/kg TS. Andre et al.^8^ studied roadside grass cuts and solid cattle manure which are sources available for dry anaerobic digestion. They determined their methane potential at laboratory scale and showed a high degree of seasonality, such as 202.9 and 167.9 Nm^3^CH_4_/tVS, respectively.

This study is about the valuation of the grass through anaerobic digestion along with cow manure. Despite the importance of the biodegradability of lignocellulose biomass, few studies have evaluated the lignocellulose biomass digestion kinetics and modeling of the process. The results were used to characterize anaerobic digestion using raw materials, investigate the quantitative relationship between the biogas potential and the organic content. Kinetic work on anaerobic co-digestion of grass with cow manure was carried out using three different models, the first-order kinetic model, the modified Gompertz model, and the Logistics model. Finally, energy production potential of grass and cow manure and an analysis of GHG emissions was carried out.

## Materials and methods

### Materials

Grass (festuca arundinacea type) was taken from the lawn mower of the university and collected from the surrounding garden by author. The grass was crushed using shredder, mixed and stored until use. Its particle size was about 0.5–2 mm. All methods were carried out in accordance with relevant guidelines and regulations. The sludge used in this study was taken fresh from the anaerobic wastewater treatment system of a yeast factory and cow manure was taken from the university farm. Operational parameters of the digester content are given in Table 1.

**Table 1:** Operational parameters.

Temperature (°C)	Digestion time (day)	Experiment no.	Feed ratio (grass/manure; %)	VS loaded (g)
35 ± 1	65	Exp1.	5	235.65
		Exp2.	10	259.25
		Exp3.	15	282.86
		Exp4.	20	306.46
		Exp5.	25	330.06

### Experimental set-up

**Figure 1:**
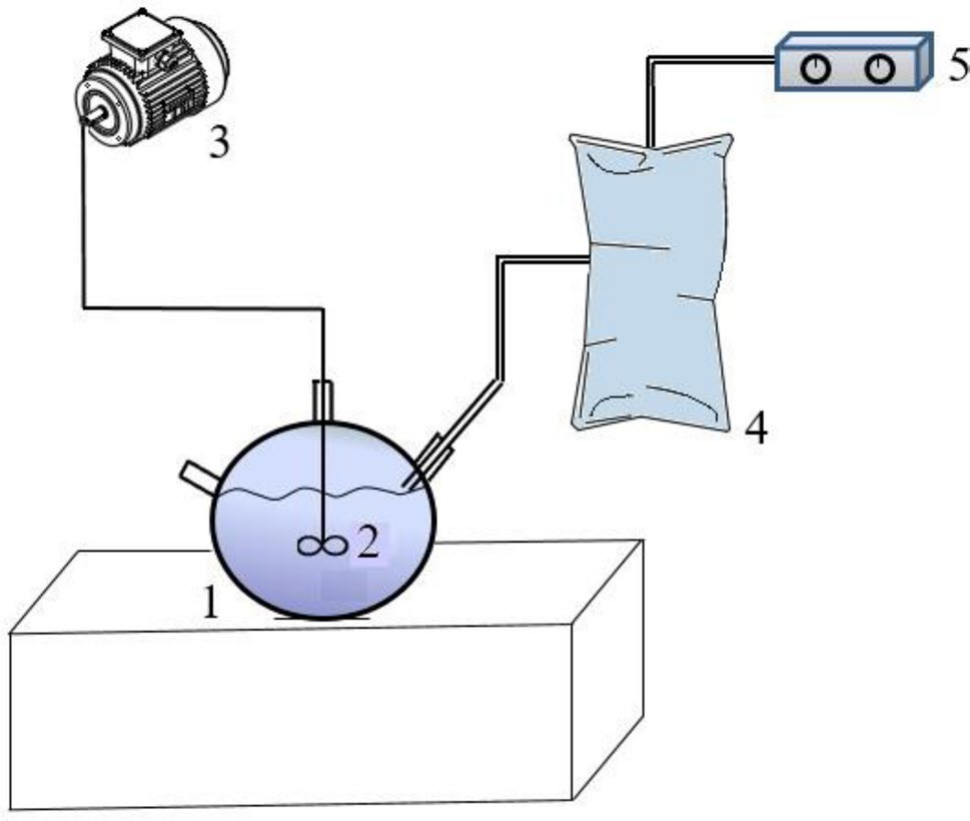
Experimental set-up. (1) Laboratory digester, (2) mixer, (3) electrical motor, (4) gas bag, (5) gas analyzer.

For experiments, 10 L laboratory scale batch glass digesters were used. The active volume of the digester was 7 L. Digesters were in a room that contain control elements for mixing and heating. Anaerobic digestion was performed at mesophilic conditions (~ 35°C), maintaining the temperature constant. Figure 1 shows the simple scheme of one digester system. Daily gas production and gas compositions were recorded everyday.

### Analysis

The properties of the raw materials can be found in Table 2. Total solids (TS) and volatile solids (VS) contents were determined by drying the substrates for 24 h at 105°C and 2 h at 550°C^21^. The analyzes were performed once daily throughout the AD procedure and were performed in duplicate. The compositions of the produced biogas were measured using an infrared gas analyzer purchased from Geotechnical Instruments, UK.

**Table 2:** Characteristics of digester contents.

Analysis (wt%)	Grass	Cow manure	Sludge
Total solid (TS)	41.73	12.75	11
Volatile solid (VS)	75.42	79.83	53.97

### Kinetic model

Three different kinetic models which are first order kinetic model, modified Gompertz model, and logistics model were applied to study the kinetics of methane production during AD of grass and cow manure. Experimental cumulative methane yield values were used in order to estimate the kinetic parameters by using nonlinear least square regression analysis. This method optimizes the value of kinetic parameters to minimize the sum of squares of differences between measured and simulated methane yield values. The applied models and equations are given as follows:

The First Order Kinetic Model

$${C}_{{\text{CH}}_{4}}={C}_{{\text{CH}}_{4}{\text{max}}}\left[1-\mathrm{exp}\left(-kt\right)\right].$$

Modified Gompertz Model:

$${C}_{{\text{CH}}_{4}}={C}_{{\text{CH}}_{4}{\text{max}}}\mathrm{exp}\left[-\mathrm{exp}\left(\frac{{r}_{{\text{CH}}_{4{\text{max}}}}\mathrm{exp}\left(1\right)}{{C}_{{\text{CH}}_{4{\text{max}}}}}\left({t}_{L}-t\right)+1\right)\right]$$

Logistic Function Model:

$${C}_{{\text{CH}}_{4}}={C}_{{\text{CH}}_{4}{\text{max}}}\left[1-\mathrm{exp}\left(-\frac{{r}_{{\text{CH}}_{4{\text{max}}}}({t}_{L}-t)}{{C}_{{\text{CH}}_{4{\text{max}}}}}\right)\right],$$

where *C*_*CH4*_ the cumulative experimental methane yield (mole/gVS); *C*_*CH4max*_ the maximum concentration of methane (mole/gVS); *k* reaction rate constant; t: digestion time (day); *r*_*CH4max*_ the maximum methane production rate (mole/gVS day); *t*_*L*_ lag phase time (day).

The validity of these models was evaluated by statistical indicators of coefficient of determination (R^2^) and root mean square error (RMSE).

## Results and discussion

### Biogas and methane production volumes and yields

**Figure 2:**
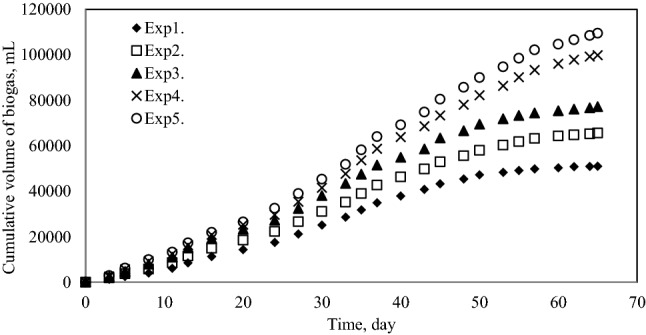
Cumulative biogas production.

**Figure 3:**
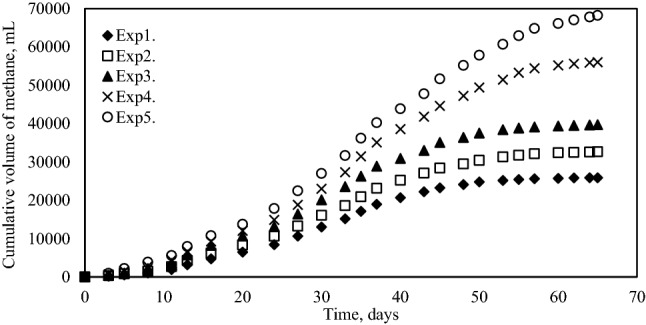
Cumulative methane production.

The cumulative volume of biogas and methane produced at different ratios was measured for 65 days during anaerobic fermentation of pretreated grass, cow manure and sludge. The results presented in Figs. 2 and 3 shows that, the Exp1. produced 51,000 mL of biogas totally and maximum biogas production was reached by Exp5. as 109,500 mL. Based on the results obtained, the maximum methane volume was also produced by Exp5. Which was about 68,000 mL.

**Figure 4:**
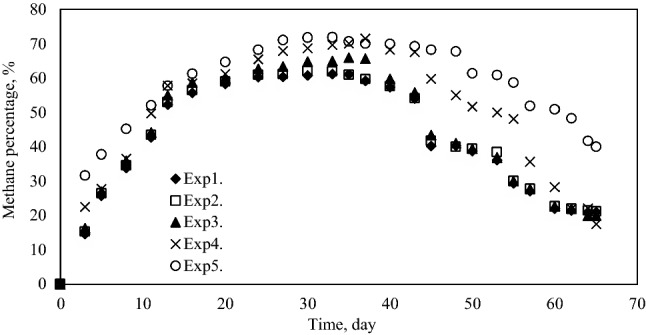
Methane percentages.

**Figure 5:**
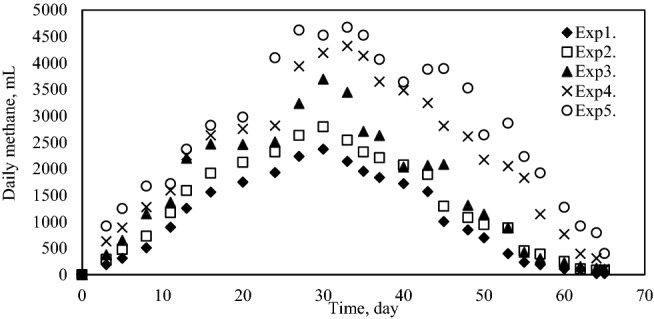
Daily methane production.

Figure 4 shows that all mixtures have high methane content (about 70%) at many points in the biogas production curve. Biogas production in all digesters occurred immediately after the first day and markedly increased for the first 40–45 days, then decreased slowly. It is seen that peak values are between 35 and 40 days since daily biogas production. The results of daily methane production are given in Fig. 5.

**Figure 6:**
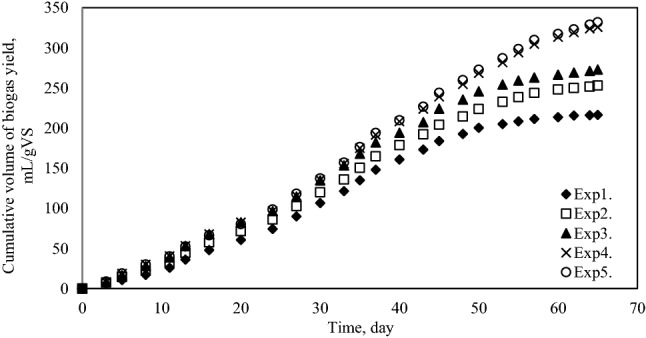
Cumulative biogas production yield.

**Figure 7:**
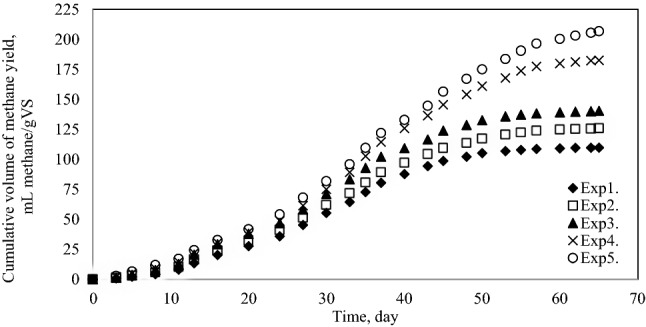
Cumulative methane production yield.

Cumulative biogas yield and methane production yield are presented in Figs. 6 and 7, respectively. The final biogas and methane yield from each mixture changes between 216.42 mL biogas/gVS; 109.73 mLCH_4_/gVS (Exp1.) and 331.75 mLbiogas/gVS; 206.64 mLCH_4_/gVS (Exp5.), respectively. Specifically, the maximum biogas and methane yield was achieved in Exp5.

The co-digestion of different mixtures of grass, cow manure and sludge compositions was carried out successfully. It depends on various important parameters of substrates as well as the process parameters of AD. Biogas production from lignocellulosic biomass is considered as an eco-friendly second-generation technology for energy production. Methane production is an efficient means of energy generation from biomass compared to other processes, exhibiting a high energy output/input ratio. Many pretreatment strategies are available for cellulosic biomass and in recent years many studies are evaluating the feasibility of these methods for accelerating the digestion process and improving biogas production from lignocellulosic biomass^22^. It can be concluded that, the amount of mixture influences the biogas and methane amounts obviously. As the grass amount increases, biogas yield was affected positively. The high lignin content in the substrates caused more total biogas yield.

### Model analysis and kinetic study

The experimental values were fitted with kinetic models in order to obtain kinetic parameters which are maximum biogas production rate, biogas yield potential and duration of the lag phase of the reaction. The parameters of kinetic models may characterize the methane production process^23,24^.

The calculated parameters of the analyzed kinetic models; the first order kinetic model, the modified Gompertz model and logistics model, are summarized in Table 3. The RMSE values was in the range of 0.827–3.384 for first order model while this value was between 0.82 and 14.3 for logistics model. It is seen from Table 3 that, the minimum RMSE values were obtained for the modified Gompertz model which were between 0.324 and 0.513.

**Table 3:** Results of kinetic study using three different models.

	First order model			Modified Gompertz model				Logistics model			
	RMSE	C_CH4max_	k	RMSE	C_CH4max_	r_CH4max_	t_L_	RMSE	C_CH4max_	r_CH4max_	t_L_
Exp1.	0.827	5.406	0.093	0.513	1.970	0.094	5	0.820	5.399	0.516	0.217
Exp2.	0.963	5.357	0.095	0.655	1.957	0.093	4.935	0.963	5.357	0.513	0.001
Exp3.	0.995	5.522	0.096	0.707	2.021	0.092	4.703	14.300	5.410	0.418	0.001
Exp4.	2.280	5.987	0.096	0.914	2.228	0.075	2.764	2.280	5.987	0.096	0.001
Exp5.	3.384	6.349	0.119	0.324	2.407	0.073	0.001	3.384	6.349	0.119	0.001

**Figure 8:**
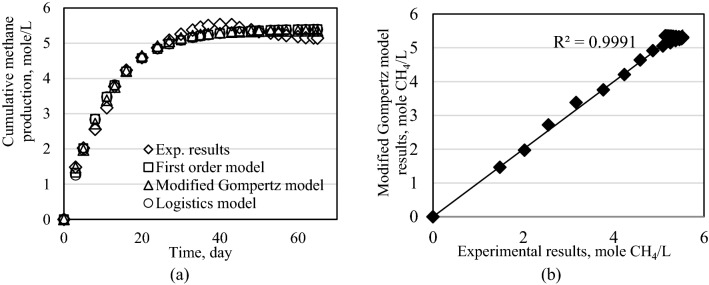
Results of models for Exp1. (**a**) Cumulative methane production for all models. (**b**) Modified Gompertz model results.

**Figure 9:**
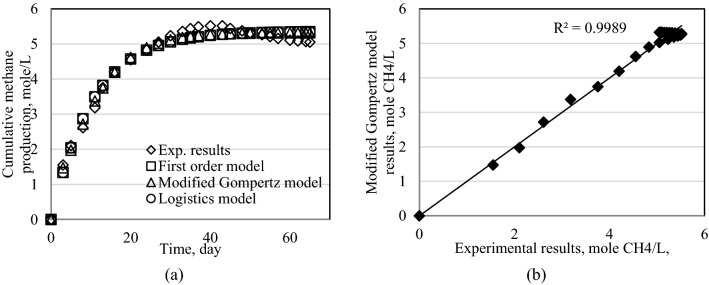
Results of models for Exp2. (**a**) Cumulative methane production for all models. (**b**) Modified Gompertz model results.

**Figure 10:**
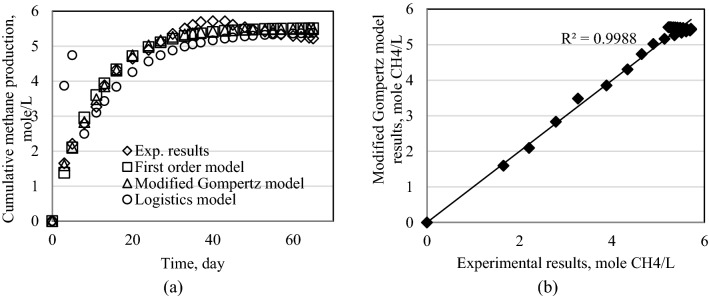
Results of models for Exp3. (**a**) Cumulative methane production for all models. (**b**) Modified Gompertz model results.

**Figure 11:**
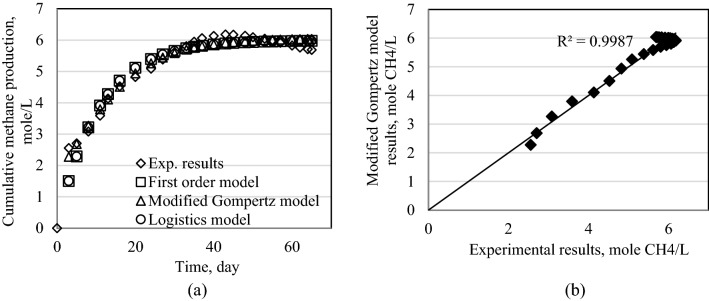
Results of models for Exp4. (**a**) Cumulative methane production for all models. (**b**) Modified Gompertz model results.

**Figure 12:**
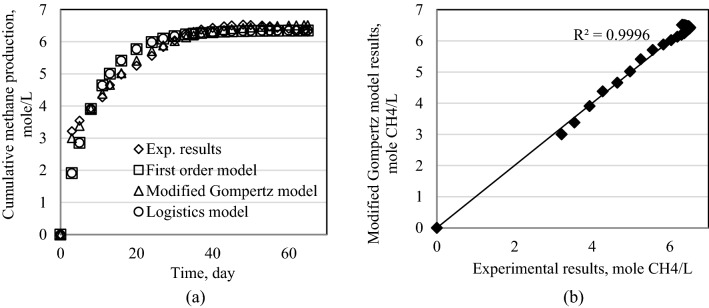
Results of models for Exp5. (**a**) Cumulative methane production for all models. (**b**) Modified Gompertz model results.

Since the minimum RMSE values were in modified Gompertz model, the estimated cumulative biogas values were plotted against the experimental values and they were given in Figs. 8, 9, 10, 11 and 12 for this model. The reliability of the model results is in reasonable agreement (R^2^ > 97%).

Several kinetic models are designed for substrates for the calculation of biogas and methane production rate. The kinetic parameters are very important to design biogas plants and to evaluate the efficieny of such plants^25^. The kinetic model studies showed that, the first order model and logistics model was not suitable for predicting biogas production because they had more fitting error values than the modified Gompertz model. All model results is in reasonable agreement value which is more than 97%. The modified Gompertz model had fitting error value below 2%.

### Greenhouse gas emission

The EU and its member states agreed to reduce their GHG emissions with 40% by 2030 compared to levels of 1990 at the COP 21 meeting in Paris in 2015^26^. The greenhouse gas emission inventory results showed that, the total GHG emissions in Turkey in 2019 is CO_2_ equivalent of 506.1 million tons. The shares of CO_2_ equivalent energy was 72% by industrial operations and product use, by 11.2% agricultural activities, by 13.4% and waste based emissions by 3.4%. On the other hand, according to the data of the year 2019, the electricity generation of the country was 304 TWh and according to these production sources, 37.1% was generated from coal, 0.1% from liquid fuels, 18.9% from natural gas, 29.2% from hydropower, and 14.7% from renewable energy and waste sources^27^.

Biogas is a a clean and sustainable energy generation option that can supply significant GHG savings compared to fossil fuels^28^. In this study, positive effects of biogas production by means of GHG by using co-digestion of cow manure and grass was studied for Turkey. Firstly the biogas and energy equivalents produced according to grass was calculated. Total municipal waste value was accessed through the formal statistical organization^27^. During the calculations, amount of grass was taken as the 7.9% of the total municipal waste^29^. Average production of 500–600 m^3^ of biogas per tons of VS could be achieved from the AD of grass^20^. But in this study this value was taken as 300 m^3^ as mentioned in reference^10^. Also, electrical energy equivalent was calculated used as 1.9–2.2 kWh/m^3^ of biogas^30^. According to International Energy Agency, the CO_2_ emission values of electricity generation when it is produced from coal is 0.98 kg/kWh; from natural gas is 0.41 kg/kWh; and from petroleum is 0.95 kg/kWh^31^. As a result of the calculations, Table 4 shows that nearly 456,000 tons of CO_2_ per year can be eliminated by converting grass into biogas.

**Table 4:** Biogas, electricity and CO_2_ emissions.

Municipal waste (tons/year)	Grass waste (tons/year)	Grass VS (tons/year)	Biogas (m^3^/year)	Electrical energy (kWh/year)	CO_2_ release petroleum equivalent (tons/year)
32,209,000	2,544,511	800,828	240,248,400	~ 480,400,000	~ 456,000

According to the results of the greenhouse gas inventory, in Turkey, the total greenhouse gas emission in 2020 increased by 3.1% compared to the previous year and was calculated as 523.9 million tons CO_2_ equivalent. The total greenhouse gas emission per capita was calculated as 4 tons of CO_2_ eq. in 1990, and 6.2 tons of CO_2_ eq. in 2019 and 6.3 tons of CO_2_ eq in 2020^27^. The greenhouse effect of electricity generation is reflected in the extent of the CO_2_ emissions, which amount to 362–891 ton CO_2_/GWh when the electricity is generated from natural gas, 547–935 ton CO_2_/GWh for electricity generated from petroleum, and 756–1372 ton CO_2_/GWh for that produced by coal^32^.

## Conclusion

In recent world, where the need for energy is increasing every day, energy production is considered to be extremely important. Since fossil sourced fuels are limited and the fact that some of the energy used in Turkey is outsourced, tendency to the renewable energy sources is considered to be extremely important. Biogas produced from lignocellulosic substrate and animal manure has the potential to be a promising renewable energy source. This study investigated the enhancement of biogas production through mesophilic co-digestion of grass and cow manure. Co-digestion yielded maximum 216.42 mLbiogas/gVS and 331.75 mLbiogas/gVS. The kinetic models, namely, first order kinetic model, modified Gompertz model and Logistics model were applied to the experimental results and the kinetic parameters were obtained for each model.

Various abatement procedures are presented to conclude that greenhouse gas emission can be significantly reduced by considering renewable sources. The technology consumes energy and increases the cost, which encourages it to choose an alternative way to reduce CO_2_ emissions. In addition, the author suggest that existing renewable sources would be a potential solution, effectively offering a significant reduction in greenhouse gas emissions. Although this research reflects the example of Turkey, direct greenhouse gas emissions from electricity generation in the energy sector can be reduced by generating electricity from organic waste all over the world.

## Data Availability

The datasets used and/or analysed during the current study available from the corresponding author on reasonable request.
